# Identification of Infectious Agents in High-Throughput Sequencing Data Sets Is Easily Achievable Using Free, Cloud-Based Bioinformatics Platforms

**DOI:** 10.1128/JCM.01386-19

**Published:** 2019-11-22

**Authors:** Joseph G. Chappell, Timothy Byaruhanga, Theocharis Tsoleridis, Jonathan K. Ball, C. Patrick McClure

**Affiliations:** a School of Life Sciences, University of Nottingham, Nottingham, United Kingdom; b Uganda Virus Research Institute, Entebbe, Uganda; c NIHR Nottingham BRC, Nottingham University Hospitals NHS Trust and the University of Nottingham, Nottingham, United Kingdom; Boston Children's Hospital

**Keywords:** high-throughput sequencing, bioinformatics, virology

## LETTER

It was with great interest that we read the recent publication by Brinkmann et al. (1) on the comparison of various methodologies for diagnosing viral infections in high-throughput sequencing (HTS) data sets. The authors demonstrated that there is a plethora of workflows and pipelines available to analyze HTS data sets and the choice of technique can lead to different results, even with a uniform proficiency testing data set.

Processing HTS data sets is computationally intensive, may require significant investment, and often necessitates a comprehensive technical background to fully analyze the results. Currently, these requirements can limit the use of HTS, preventing clinicians and researchers with minimal funding or expertise in bioinformatics from exploring and exploiting this powerful technology.

However, several online tools, such as IDseq (2, 3) and Genome Detective (4), have recently been made available for research involving pathogen discovery and identification. The cloud-based nature of these tools removes the requirement for users to have high-specification computers for data processing, and automated identification of microbial sequences reduces the need for any significant background in bioinformatics. HTS data sets, with identifying information removed, are simply uploaded, and annotated sequence matches to potential pathogens are delivered within hours, in a format that can be easily interpreted by those with relevant clinical or academic skills. While IDseq automatically discards any human genomic reads, the submission of data sets containing patient sequences, although anonymized, to third-party platforms necessitates ethical consideration and permission.

We evaluated IDseq and Genome Detective against the simulated *in silico* data set provided by Brinkmann et al. (1). IDseq analysis took 92 min from the initiation of sample uploading to the presentation of the mapped reads, one-half of the time for the fastest participant (participant 1) reported by Brinkmann et al. (1). Of the 6,339,908 reads in the data set, 1,362,725 reads (21.5%) passed host filtering; of those, 996,855 reads (73.2%) mapped to bacterial nucleotide databases (70.3% to nonredundant protein databases). Genome Detective identified and removed 6,290,069 reads (99%) as nonviral hits, completing the analysis in only 16 min. Both platforms detected all four viruses in the data set (Table 1). Detection of Torque teno virus, human herpesvirus 1, and measles virus was not as sensitive as in many of the other participant workflows. However, both IDseq and Genome Detective identified the highly divergent avian orthobornavirus (55% similarity to a reference sequence), whereas 9 of the 13 workflows in the study by Brinkmann et al. (1) did not.

**TABLE 1:** Comparison of viral reads identified by IDseq and Genome Detective and the COMPARE virus proficiency tests^*a*^

Method and database	Sensitivity (%)				Time (h)
	Torque teno virus	Human herpesvirus 1	Measles virus	Avian bornavirus	
Proficiency test, participants 1 to 13 (median [range])	100 (0–102)	99 (10–400)	100 (0–140)	0 (0–100)	15.5 (3–216)
IDseq					
Nucleotide	59	56	69	0	1.5
Nonredundant protein	59	55	70	53	
Genome Detective	100	84	82	41	0.25

a A sensitivity of >100% indicated false-positive results.

Our results show that both platforms can accurately identify viral genomes in HTS data sets, with little or no prior knowledge of bioinformatic approaches. IDseq has the additional capability to detect bacterial genomes as well as viral genomes. While not as sensitive as some of the other methodologies tested, IDseq and Genome Detective were able to identify all of the infectious agents included in the proficiency data set, in a fraction of the time reported for the other pipelines, and required very little local computational power. IDseq, Genome Detective, and similar free cloud-based online tools will significantly reduce the barrier to entry for exploiting HTS, without the hardware and background required for traditional bioinformatics approaches.
